# GIScaSpA—study of subclinical gut involvement in axial spondyloarthritis

**DOI:** 10.1093/rap/rkae016

**Published:** 2024-02-06

**Authors:** Carolina Mazeda, Susana P Silva, C Pinto Oliveira, Sofia Azevedo, Anabela Barcelos

**Affiliations:** Rheumatology Department, Centro Hospitalar do Baixo Vouga, Aveiro, Portugal; Centro Académico Clínico Egas Moniz, Health Alliance, Aveiro, Portugal; EpiDoc Unit, Nova Medical School, NOVA University Lisbon, Lisbon, Portugal; Rheumatology Department, Centro Hospitalar do Baixo Vouga, Aveiro, Portugal; Centro Académico Clínico Egas Moniz, Health Alliance, Aveiro, Portugal; Rheumatology Department, Centro Hospitalar do Baixo Vouga, Aveiro, Portugal; Centro Académico Clínico Egas Moniz, Health Alliance, Aveiro, Portugal; Rheumatology Department, Centro Hospitalar do Baixo Vouga, Aveiro, Portugal; Centro Académico Clínico Egas Moniz, Health Alliance, Aveiro, Portugal; Rheumatology Department, Centro Hospitalar do Baixo Vouga, Aveiro, Portugal; Centro Académico Clínico Egas Moniz, Health Alliance, Aveiro, Portugal; EpiDoc Unit, Nova Medical School, NOVA University Lisbon, Lisbon, Portugal; Comprehensive Health Research Center, Universidade NOVA de Lisboa, Lisbon, Portugal

Key message

- Faecal calprotectin may be useful in identifying axSpA patients with subclinical bowel inflammation who might benefit from additional invasive studies.

Dear Editor, axSpA has a predilection for the axial skeleton and belongs to the group of SpAs, a set of inflammatory rheumatic diseases with common clinical, radiologic and serologic features in which extra-articular manifestations are frequent, including IBD [1]. The link between SpA and IBD has been recognized for decades, with 6–14% of axSpA patients suffering from concurrent IBD [2, 3]. Emerging evidence suggests that subclinical gut inflammation in patients with SpA is an important pathophysiological event that plays a role in disease pathogenesis. However, it is difficult to detect subclinical gut inflammation since it is largely asymptomatic.

Faecal calprotectin (FC) is a very sensitive marker for inflammation in the gastrointestinal tract. The role of FC in patients with SpA is not clearly defined [4], but some studies suggest that FC levels may predict the onset of IBD and may be related to disease activity [5–8]. The aim of our study was to test the validity of FC as a marker of intestinal inflammation in patients with axSpA and to investigate factors associated with increased FC levels in these patients.

We performed a cross-sectional study that included patients fulfilling the Assessment of SpondyloArthritis international Society (ASAS) classification criteria for axSpA, divided into two groups: AS and non-radiographic axSpA (nr-axSpA); a control group without rheumatologic diseases or IBD was also included. Individuals who were <18 years of age; had a diagnosis of another inflammatory rheumatic disease or IBD; had a history of colorectal carcinoma, infectious gastroenteritis, intestinal diverticular disease, cystic fibrosis or coeliac disease; or an impossibility of suspending NSAIDs were excluded. All patients and controls answered a structured questionnaire about the presence of gastrointestinal symptoms (the Red Flags questionnaire for IBD) and underwent FC measurement with ELISA (≥50 mg/kg was considered positive). Patients treated with oral NSAIDs underwent adequate washout (at least 3 weeks). Demographic and clinical data, alongside standard outcome measures for axSpA, including the BASDAI, BASFI, BASMI and Ankylosing Spondylitis Disease Activity Score using CRP (ASDAS-CRP) were collected. Descriptive and univariate analyses were conducted using SPSS version 25 (IBM, Armonk, NY, USA). *P*-values ≤0.05 were considered statistically significant. For the axSpA outcome measures, differences between patients with elevated *vs* normal FC levels were analysed by Student’s *t*-test and analysis of variance, adjusting for sex, age and ongoing treatment. We included 64 patients (34 with AS and 30 with nr-axSpA) and 25 controls. Demographic and clinical data are presented in Table 1.

**Table 1.** Characteristics of the population according to axSpA groups and controls

Characteristics	AS (*n* = 34)	axSpA (*n* = 30)	Controls (*n* = 25)	*P*-value^a^
Age, years, mean (s.d.)	55.5 (14.7)	44.9 (13.3)	40.2 (14.0)	**0.004**
Sex (female/male), *n*/*n*	15/19	18/12	13/11	0.205
Duration of disease, years, mean (s.d.)	11.8 (7.7)	5.4 (3.3)	–	**<0.001**
Comorbidities, *n* (%)				
Arterial hypertension	13 (38.2)	10 (33.3)	1 (4)	0.166
Dyslipidaemia	8 (23.5)	7 (23.3)	2 (8)	0.985
Diabetes mellitus	3 (8.8)	2 (6.7)	0	0.748
Obesity	1 (2.9)	1 (3.3)	0	
Smoker, *n* (%)	4 (11.8)	1 (3.3)	1 (4)	0.850
Peripheral involvement, *n* (%)	9 (26.5)	5 (16.7)	–	0.344
Dactylitis (ever), *n* (%)	0	1(3.3)	–	0.283
Enthesitis (ever), *n* (%)	1 (2.9)	2 (6.7)	–	0.482
Uveitis (ever), *n* (%)	9 (26.5)	4 (13.3)	–	0.192
HLA-B27 positive, *n* (%)	25 (73.5)	13 (43.3)	–	**0.014**
BASDAI, mean (s.d.)	3.4 (2.5)	3.8 (2.4)	–	0.589
BASFI, mean (s.d.)	3.3 (2.5)	3.3 (2.1)	–	0.981
ASDAS-CRP, mean (s.d.)	2.3 (1.0)	2.4 (1.1)	–	0.070
BASMI, mean (s.d.)	4.2 (1.8)	3.1 (1.7)	–	0.226
Therapy, *n* (%)			–	
NSAIDs^b^	16 (47.1)	14 (46.7)		0.124
cDMARDs	2 (5.9)	2 (6.7)		0.897
bDMARDs	9 (26.5)	11 (36.7)		0.408

NSAID: nonsteroidal anti-inflammatory drugs ; bDMARD: biologic DMARD; cDMARD: conventional DMARD.

Significant values in bold.

a Comparison between nr-axSpA and AS groups.

b Washout before collecting stool.

In the AS group, 2.9% had nocturnal diarrhoea, a first-degree relative with confirmed IBD and rectal urgency; in the nr-axSpA group, 6.7% had abdominal pain 30–45 min after meals and 3.3% had chronic abdominal pain; all controls were asymptomatic. Elevated FC was observed in 32.4% of AS patients [129.2 (s.d. 404.0)], 23.3% of nr-axSpA patients [70.9 (s.d. 114.4)] and 4.2% of controls [21.9 (s.d. 18.4)]. FC was significantly higher in each axSpA subtype *vs* controls (*P* = 0.03). There were no statistically significant differences in ASDAS, BASDAI, BASFI or BASMI scores between axSpA patients with normal *vs* high FC levels. Several studies suggest that a significant number of axSpA patients exhibit asymptomatic intestinal inflammation, with FC playing a crucial role as a marker for this microscopic intestinal inflammation (Supplementary Table S1, available at *Rheumatology Advances in Practice* online). The percentage of increased FC ranged from 29 to 74.5%, with higher values observed in patients with AS, significantly differing from controls, which aligns with our study. Despite elevated FC levels, the majority of patients remain asymptomatic. Significant heterogeneity was observed among various studies regarding the relationship between FC and different disease activity indices. Therefore, larger population studies and invasive tests are necessary to evaluate the potential impact this marker may have on managing this condition.

## Supplementary Material

rkae016_Supplementary_Data

## Data Availability

The data that support the findings of this study are available from the corresponding author upon reasonable request.
