# Supplementary material for: GIScaSpA—study of subclinical gut involvement in axial spondyloarthritis
Source: Rheumatol Adv Pract. 2024 Feb 6;8(1):rkae016. doi: 10.1093/rap/rkae016 (PMC10898325; doi:10.1093/rap/rkae016)
Supplement: rkae016_Supplementary_Data [file rkae016_supplementary_data.docx]

**Supplementary Table S1** - Studies investigating the role of fecal calprotectin in patients with axSpA.

| **Ref (), Year** | **Country** | **Disease** | **Number of cases** | **Number of controls** | **Normal FC values, mg/kg** | **Patients with elevated FC** | **Controls with elevated FC** | **Gastrointestinal symptoms** | **Correlation with disease activity** |
| --- | --- | --- | --- | --- | --- | --- | --- | --- | --- |
| Klingberg E et al (3), 2012 | Sweden | AS | 205 | 80 | <50 | 68% | NS | No association | CRP, ESR, ASDAS |
| Matzkies F et al (4), 2012 | USA | AS | 39 | 42 | <50 | 41% | 10% | NS | BASFI |
| Duran A et al (5), 2016 | Turkey | AS | 51 | 43 | <50 | 74.5% | 30.2% | NS | CRP, ESR. BASFI, BASDAI |
| Klingberg E et al (6), 2017 | Sweden | AS and nr-axSpA | 164 | NS | <50 | 63.4% | NS | No association | CRP, ESR, ASDAS-CRP, BASDAI, BASFI and BASMI |
| Østgård RD et al (7), 2017 | Denmark | AS and nr-axSpA | 30 | NS | <50 | 50% | NS | NS | NS |
| Olofsson T et al (8), 2019 | Sweden | AS and nr-axSpA | 130 | 35 | <50 | 27% in nr-axSpA/38% in AS | 6% | No association | ASDAS-CRP, BASFI |

Abbreviation: FC - fecal calprotectin; AS - ankylosing spondylitis; nr-axSpA - non-radiographic axSpA; NS, non-specified; n- number; CRP - C-reactive protein; ; ESR - erythrocyte sedimentation rate; ASDAS - Ankylosing Spondylitis Disease Activity Score; BASDAI - Bath Ankylosing Spondylitis Disease Activity Index; BASFI - Bath Ankylosing Spondylitis Functional Index; BASMI - Bath Ankylosing Spondylitis Metrology Index.
